# Automated quantification of central corneal epithelial thickness for the diagnosis of limbal stem cell deficiency

**DOI:** 10.1371/journal.pone.0358258

**Published:** 2026-10-05

**Authors:** Micah Vinet, Nathan Siu, Clemence Bonnet, Craig Choi, Sarah Wu, Celso Nguyen, Corey Arnold, William Speier, Sophie X. Deng

**Affiliations:** 1 Jules Stein Eye Institute, University of California Los Angeles, Los Angeles, California, United States of America; 2 Department of Bioengineering, University of California Los Angeles, Los Angeles, California, United States of America; 3 Biomedical AI Research Lab, University of California Los Angeles, Los Angeles, California, United States of America; 4 Medical Informatics Home Area, Graduate Programs in Bioscience, UCLA, Los Angeles, California, United States of America; 5 Department of Computational and Systems Biology, Graduate Programs in Bioscience, UCLA, Los Angeles, California, United States of America; 6 Department of Molecular and Cell Biology, University of California, Berkeley, California, United States of America; 7 Department of Radiology, David Geffen School of Medicine, University of California Los Angeles, Los Angeles, California, United States of America; Save Sight Institute, AUSTRALIA

## Abstract

Limbal Stem Cell Deficiency (LSCD) is a corneal disorder characterized by conjunctivalization of the cornea. Central corneal epithelial thickness, measured using Anterior Segment Optical Coherence Tomography (AS-OCT), is a biomarker for LSCD. Manual measurement of epithelial thickness remains the gold standard for diagnosis because existing automated commercial software is unreliable in severe cases. Standard machine learning approaches have limited applicability for automating this measurement due to the difficulty of distinguishing corneal epithelium from scar tissue, and imprecise clinical labels. The goal of this study is to develop a deep learning pipeline to automate measurement of central epithelial thickness in AS-OCT images of eyes with LSCD. To accomplish this goal, we developed AI-CE, a model based on Multi-scale U-Net (MSU-Net) trained to accurately delineate the epithelial layer. The primary dataset comprised of 134 patient eyes, including 47 healthy normal eyes (35.0%), 34 with Stage 1 (25.4%), 34 with Stage 2 (25.4%), and 19 with Stage 3 (14.2%) LSCD. Performance was evaluated by calculating the average mean absolute error between model segmentations and manually measured clinical assessments. AI-CE model performance was compared against a commercially available segmentation software to demonstrate its improvement over the current state-of-the-art. The proposed model achieved a mean absolute error of 5.10 μm, significantly improving (p < 0.01) over clinical software (12.20 μm). This result was verified in a secondary retrospective dataset which similarly showed significant improvement over commercial software. We also demonstrate that clinical epithelial thickness values provided by AI-CE can distinguish between LSCD stages similarly to manual evaluations.

## Introduction

Limbal stem cell deficiency (LSCD) is a corneal disease characterized by the replacement of corneal epithelium by conjunctival epithelium due to loss of limbal stem cells (LSCs) and/or their niche [1]. Corneal epithelial thickness (CET) in the central cornea has been identified as a biomarker of LSC function [2]. Clinically, CET is estimated from anterior segment optical coherence tomography (AS-OCT), a widely accessible and non-contact imaging modality [2] that can allow visualization of corneal layers with up to 2–3 micrometer (μm) resolution [1]. This assessment involves trained experts manually measuring the thickness, a process that is time consuming and subject to significant inter- and intra-rater variability.

Previous studies have shown that decreasing CET correlates with LSCD disease severity [1–5]. Automated epithelial layer measurement can be performed by commercial software (Optovue RTVue XR 100 Avanti, Optovue Inc., Fremont, CA) using demarcation of the anterior epithelial and Bowman’s layers. These commercial algorithms have been approved by the FDA for the measurement of epithelial, stromal, and corneal thickness [6]. In images with LSCD and stromal scars, however, this commercial software often fails to generate correct segmentations, which can lead to unreliable evaluations of corneal epithelial thickness [7]. The difficulty of evaluating LSCD within commercial software has been attributed to stark differences in CET reflectivity, in many cases due to subepithelial scarring (typically hyperreflective) associated with LSCD compared to normal CET reflectivity (typically hyporeflective) [2,3]. Therefore, manual measurements remain the clinical standard for assessing CET for LSCD diagnosis [1–3].

An automated method for estimating CET could potentially reduce the workload of ophthalmologists, improve diagnosis efficiency, and improve intra- and inter-reader reliability. However, this clinical problem poses several challenges that prevent traditional application of deep learning (DL) segmentation methods. First, the current clinical protocol for evaluating LSCD in AS-OCT images involves scalar CET measurement values, rather than producing the segmentation masks required by most segmentation models to meaningfully represent relevant image features in latent space (the compressed internal representation where a model encodes image structure) [8–10]. These measurements are recorded at the patient level, so they do not capture variation across the corneal surface, nor do they specify the locations within the image where they were recorded. Second, in many Stage 3 (high disease severity) images, there are regions of healthy epithelium directly adjacent to subepithelial scar, with boundaries that exist as ambiguous gradients, which can cause difficulty when attempting to detect a boundary during inference [11–13]. Third, the epithelium can have variable localization, shape, and thickness as LSCD severity progresses. In particular, subepithelial scarring in severe LSCD cases often drastically alters the epithelial morphology, causing it to deviate significantly from consistent patterns observed in earlier stages. These regions of interest are difficult to capture for a DL model, as learned representations of severe LSCD epithelium may contrast with those of epithelium in less severe LSCD images [14–16].

The goal of this project is to create a novel pipeline for segmenting the corneal epithelium and measuring CET (AI-CE) using only scalar clinical measurements (single-number values rather than pixel-level annotations) as labels. This pipeline consists of three main stages: 1) automatic generation of approximate training masks using edge detection guided by clinical measurements; 2) an informed patching method guided by the corneal surface; and 3) patch-level segmentation of the corneal epithelium with image-level reconstruction and thickness measurement. The performance of this pipeline is tested against a standard full image segmentation model as well as FDA approved clinical software designed for this task. We demonstrate that this pipeline significantly outperforms existing methods, providing accurate epithelial segmentations and CET measurements that can address limitations in the current clinical pipeline.

### Related work

The field of OCT and AS-OCT image segmentation has broadened in recent years. Most existing models have used a UNet [17] as a backbone, including CorneaNet by dos Santos et al. [18], a model that segmented all layers of the cornea individually within healthy and keratoconus images. A model by Pham et al. [19] similarly localized various structures of the anterior segment in AS-OCT images, not including the epithelial layer. Li et al. [20] took a multitask learning approach for all broad structures within AS-OCT images, although they did not segment the epithelial layer individually. Goyanes et al. [21] performed AS-OCT segmentation of the ciliary muscle using a feature pyramid network architecture. SeqCorr-EUNet by Fang et al. [22] focused on segmenting the entirety of the cornea, but not individual corneal layers, using a dual-flow architecture. Another study by Win et al. [23] performed segmentation of all layers of the cornea using a graph-based approach, but did not include LSCD images. These models do not focus on segmentation of epithelial regions affected by subepithelial scarring, which is required to correctly measure CET in LSCD images due to variability in reflectivity, and thus they are not applicable to this study.

There have also been studies that focus on understanding underlying image features of the epithelial layer within AS-OCT images. Elsawy et al. created a graph-based gradient edge-cost algorithm that measured the thickness in pathological, but not LSCD, corneas [24]. A study by Levy et al. analyzed patterns within epithelial maps for various eye diseases, including LSCD, but did not measure epithelial thickness [7]. Another study, by Goto et al., used Fourier harmonic analysis to analyze corneal shape irregularity in LSCD, but did not discuss measurement of CET [25]. Finally, a study by Greenfield et al. showed potential in detecting ocular surface squamous neoplasia, a disease with abnormal epithelial layer, using a vision transformer DL model [26]. While these studies focused on quantifying image features of the epithelial layer, in some cases for LSCD images, no prior study has examined segmentation of AS-OCT images for classifying LSCD severity by measuring CET using DL methods [2,5].

## Materials and methods

### Dataset

The AS-OCT scans used in this study were acquired at the Stein Eye Institute after the approval of the Institutional Review Board at the University of California, Los Angeles (UCLA, IRB #24–1047). Patients with normal, healthy eyes in the dataset had written consent obtained. The diseased eyes in the dataset had verbal consent obtained as the AS-OCT scans were a standard of care. Consent was witnessed and documented by clinical cornea specialists during data collection and was consistent with the protocol for the study as specified by the IRB. The study adhered to HIPAA regulations and complied with the Declaration of Helsinki. All data was accessed for research purposes following IRB approval on November 25th, 2024. All patient information was de-identified during initial data collection. The main dataset utilized for training and testing the AI-CE model consisted of 134 patients (134 eyes) using an Optovue RTVue XR 100 Avanti Edition scanner. In this dataset, 3 scans per eye were taken, representing 402 scans.

As part of their clinical evaluation, each patient’s LSCD severity was staged using CET in combination with parameters obtained from other imaging modalities (slit-lamp biomicroscopy and in vivo laser scanning confocal microscopy). This staging categorized the severity for each of the eyes as either normal (n = 141 scans) or one of three LSCD stages: Stage 1 (n = 102 scans), Stage 2 (n = 102 scans), or Stage 3 (n = 57 scans) [1,2]. Each stage was graded via a clinical score based on the global consensus of the stages of LSCD [1,2].

A secondary retrospective dataset including 12 patients (24 eyes) was collected from patients with one normal eye and one Stage 3 LSCD eye, representing an additional 72 scans [27]. This secondary dataset was used as a held-out test set to further evaluate the AI-CE model.

### Clinical labels

The labels for both datasets are a set of manually measured CET values measured by cornea specialists (M-CET) for each individual AS-OCT scan. These values were measured following a consistent clinical protocol: using one image per subject, specialists took one measurement in the central cornea, and two additional measurements 1 mm apart on either side. The average of these three measurements was used clinically for diagnosing each patient, and thus were also used in this study as the ground truth label for each AS-OCT scan for that patient. In the main dataset, normal eyes had a median measurement of 55 μm (range: 46–60 μm), Stage 1 eyes had a median of 43 μm (range: 31–62 μm), Stage 2 eyes had a median of 36 μm (range: 0–61 μm), and Stage 3 eyes had a median of 0 μm (range: 0–27 μm). In the secondary dataset, normal eyes had a median measurement of 53 μm (range: 41–61 μm) and Stage 3 eyes had a median measurement of 24 μm (range: 0–47 μm). This labeling was manually and independently quantified by two senior cornea specialists, with an inter observer variability of <10%, confirming high inter-observer agreement.

For each Stage 3 scan in the main dataset, a binary label of subepithelial scar presence was created by cornea specialists via visual interpretation of each scan. There existed 39 scans with subepithelial scarring, and 18 scans without.

### Mask creation

Traditionally, DL image analysis models require pixel level labels created from manual segmentations as inputs for training. In this case, only scalar M-CET measurement values were available for each of the subjects. Thus, it was necessary to generate training masks from the raw AS-OCT images to train the DL model. To create these masks, an initial edge detection of the top of the cornea, *C*, was performed in each image using a pre-trained diffusion-based edge detection model [28]. This step was followed by additional image preprocessing steps, including morphological smoothing, before shifting the top line down to estimate the bottom of the epithelium. This shift was performed for each pixel along the horizontal axis of the image, within a range of 30 μm above and below the M-CET measurement label, $x^{\prime }$. The offset of the bottom line, *x*, was chosen to be the line within this range, $x\in [x^{\prime }+30\mu m,x^{\prime }-30\mu m]$, that had the maximal sum of intensity values of the Laplacian of Gaussian ($\nabla^{2}G$) filtered AS-OCT image (*I*):


$$\begin{array}{c} \underset{x}{\text{arg max}}\int_{C(x)}\left((\nabla^{2}G)*I\right)\;ds \end{array}$$ (1)

where *C*(*x*) is the top corneal contour shifted vertically down by an offset *x*, *ds* indicates that the integral accumulates filtered intensity along the length of that contour, and $\text{arg}\max_{x}$ selects the offset *x* producing the maximum accumulated value.

This approach resulted in an approximate contour of the bottom of the epithelial layer. Images containing subepithelial scarring, based on the clinical binary labels, were given blank segmentation masks to aid the AI-CE model in learning the difference in reflectivity between healthy and subepithelial scar regions.

### Corneal surface-guided patching

Patching was chosen to train the AI-CE model because the epithelial layer is a disproportionately small portion of the corneal image. To create patches for training, once the top and bottom of the mask locations were established, patches were generated along the top layer of the mask (Fig 1). The center of each of the patches was sampled along the top of the segmentation. The patches were defined so that the horizontal edges were parallel to the tangent of the top of the epithelial layer. A 50% overlap stride in the direction parallel to the epithelial layer was performed between the patches to provide a greater number of training patches for input into the AI-CE model.

**Fig 1:**
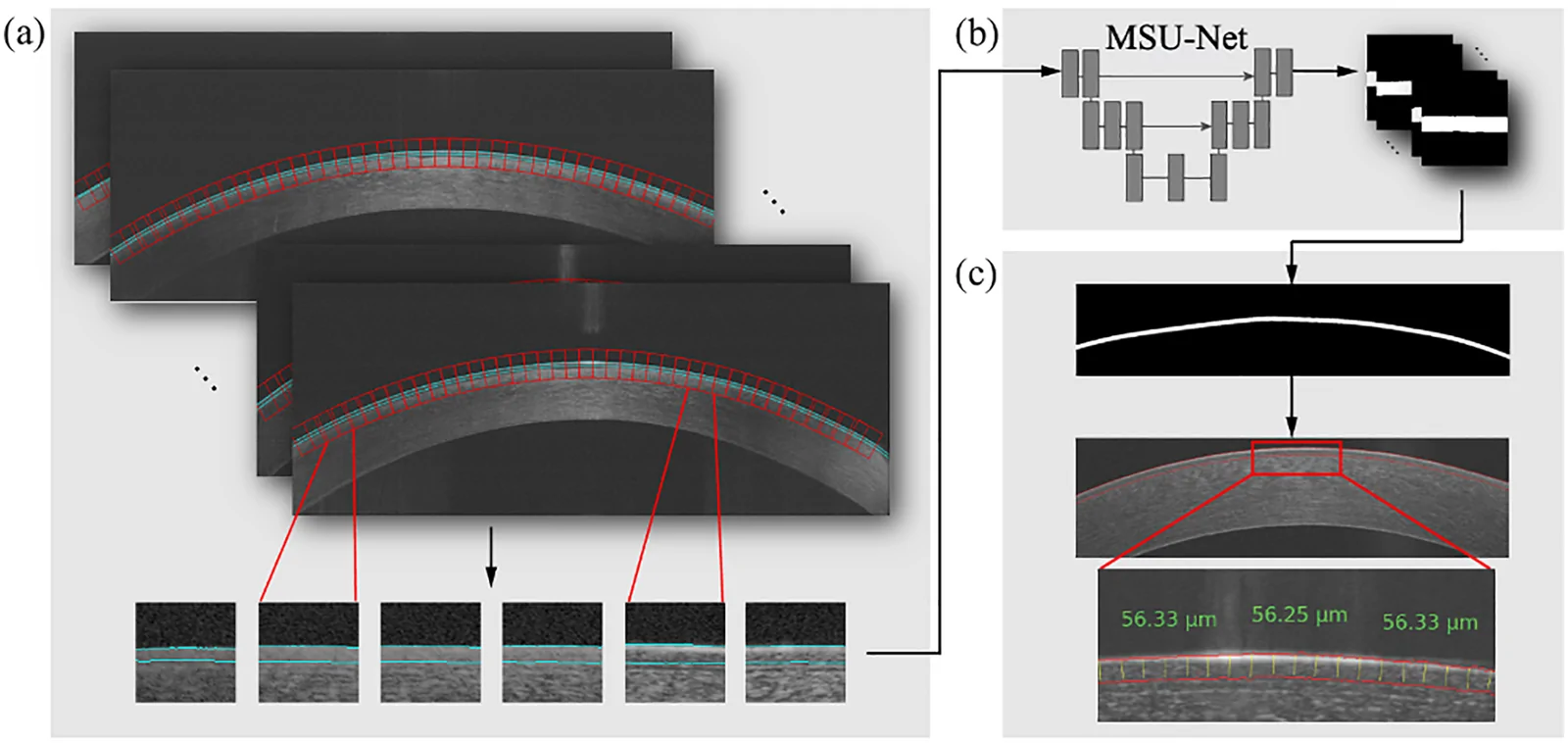
Overview of the methods of the pipeline. AS-OCT images are patched along the epithelial layer (a), are used as inputs to train and test the Multi-scale U-Net (MSU-Net) model (b), before the output patches are used to reconstruct the original full-size images and measure the predicted CET (c).

### Model training and mask reconstruction

For the AI-CE model, Multi-scale U-Net (MSU-Net) [29] architecture was trained to segment the top and bottom of the epithelial layer. The AI-CE model was trained with a five-fold cross validation, where each individual fold contained a different 70% train, 10% validation, and 20% test split such that all images from a given subject were in the same split. The dataset was stratified to ensure each of the five folds contained the same distribution across LSCD stages. For each fold, the model was trained for 100 epochs—a single epoch being one complete pass through the training and validation data—with Adam optimization, a learning rate of 1e-3, and a batch size of 32. Dice loss was used for training the model and resulted in near identical loss curves across all folds. Dice loss measures the overlap between the predicted segmentation and the target mask, and is defined as:


$$\begin{array}{c} {ℒ}_{\text{Dice}}=1-\frac{2\sum_{i=1}^{N}p_{i}g_{i}+\epsilon }{\sum_{i=1}^{N}p_{i}+\sum_{i=1}^{N}g_{i}+\epsilon }, \end{array}$$ (2)

where $p_{i}\in [0,1]$ is the predicted probability that pixel *i* belongs to the epithelium, $g_{i}\in [0,1]$ is the corresponding value in the target mask, *N* is the total number of pixels, and ϵ is a small constant added for numerical stability. A loss of 0 indicates perfect overlap, while a loss of 1 indicates no overlap.

The images of the cornea were reconstructed by aligning the predicted mask patches based on their original position in the image. The union of all the predicted patch masks were combined to create the full predicted epithelial layer for each image. The CET calculation for each image was performed by calculating the average distance between the top and bottom of the epithelial layer along lines perpendicular to the surface segmentation across the entire image, denoted AI-CET.

To confirm that the patching approach was an improvement, a baseline standard MSU-Net model was trained using original AS-OCT images, with the same five-fold train/validation/test splits, training model parameters, and downstream evaluation as the AI-CE model.

## Results

Across the entire main dataset, the MAE of the AI-CE model (5.10 μm) was significantly (p < 0.001) lower than OCT-CET (12.20 μm) (Table 1). AI-CE had a significant overall Pearson correlation with M-CET (r = 0.94, p < 0.01), while OCT-CET did not (r = 0.32, p = 0.07) (Fig 2). AI-CE had a significantly lower MAE for stage 1, 2, and 3 LSCD (4.54 μm, 5.18 μm, 10.41 μm, respectively; p < 0.001 for each) and higher correlation with M-CET (r = 0.67, r = 0.89, r = 0.10, respectively; p < 0.001 for each) compared to OCT-CET (MAE = 8.45 μm, MAE = 10.20 μm, MAE = 46.07 μm, and r = 0.41, r = 0.18, r = 0.01, respectively). While AI-CET had slightly higher MAE and lower correlation for normal (MAE = 3.40 μm, r = 0.45) compared to OCT-CET (MAE = 2.03, r = 0.67), the difference was not statistically significant (p = 0.18) (Fig 3).

**Table 1:** Result metrics (MAE and Pearson r value) across all, and across the individual normal and three LSCD severity classes (Stage 1, Stage 2, and Stage 3) for the AI-CE, MSU-Net, and OCT-CET models tested on the original dataset.

Class	MAE (μm)					Pearson Correlation				
	All	Normal	Stage 1	Stage 2	Stage 3	All	Normal	Stage 1	Stage 2	Stage 3
**AI-CE**	**5.10 ± 4.61***	3.40 ± 1.88	**4.54 ± 2.95***	**5.18 ± 3.17***	**10.41 ± 8.06***	0.94*	0.45	0.67*	0.89*	0.10*
**MSU-Net**	7.20 ± 6.04	3.86 ± 2.75	5.03 ± 3.57	6.14 ± 5.45	15.43 ± 9.74	0.89	0.43	0.34	0.65	0.04
**OCT-CET**	12.20 ± 8.36	**2.03 ± 1.36**	8.45 ± 3.98	10.20 ± 5.80	46.07 ± 10.09	0.32	**0.67**	0.41	0.18	0.01

Note: *p < 0.01.

**Fig 2:**
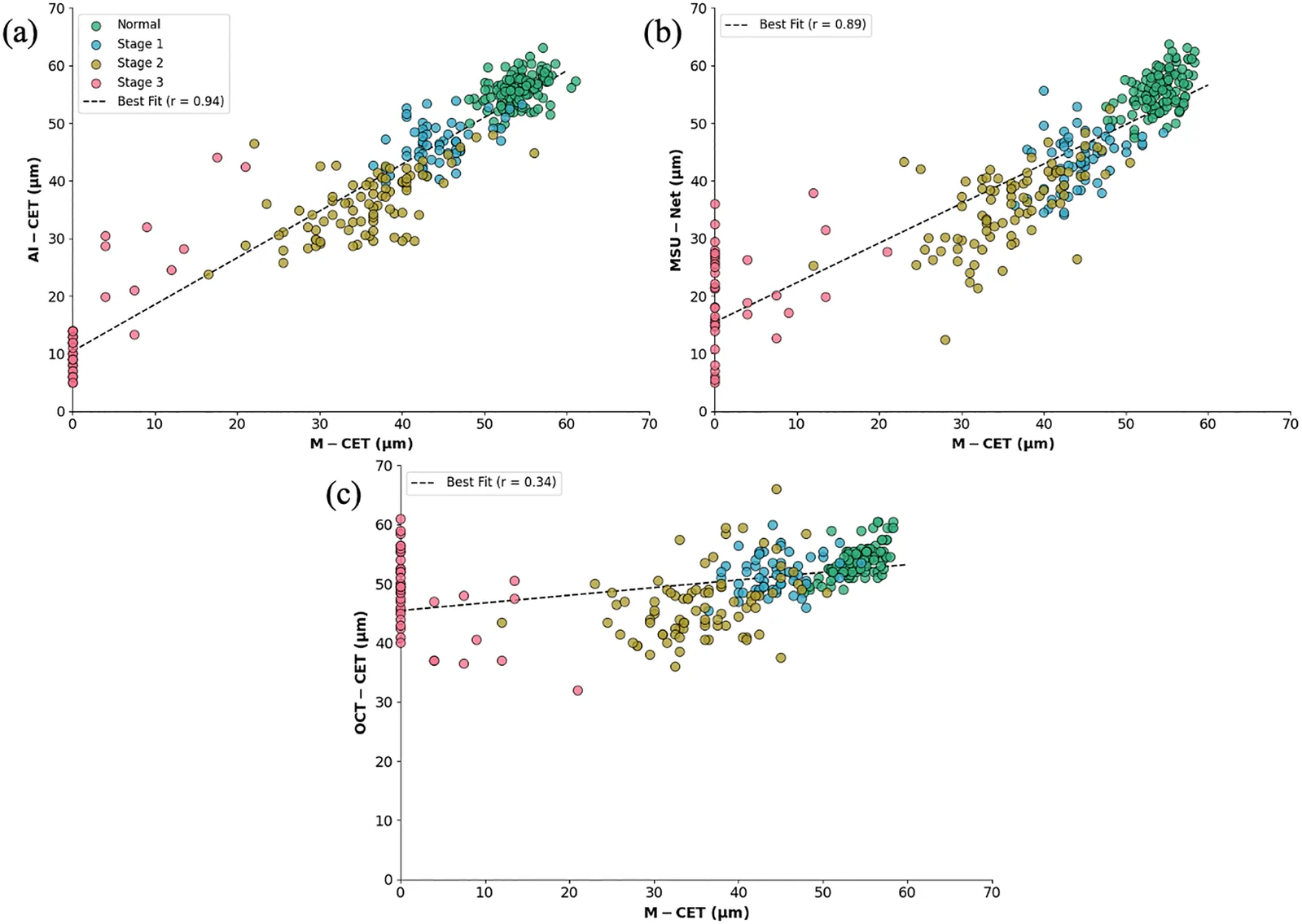
Primary dataset correlations. Correlations between the manually measured epithelial thickness values (M-CET) with those automatically calculated using (a) the proposed model (AI-CET), (b) the baseline (MSU-Net), and (c) the current commercially available solution (OCT-CET).

**Fig 3:**
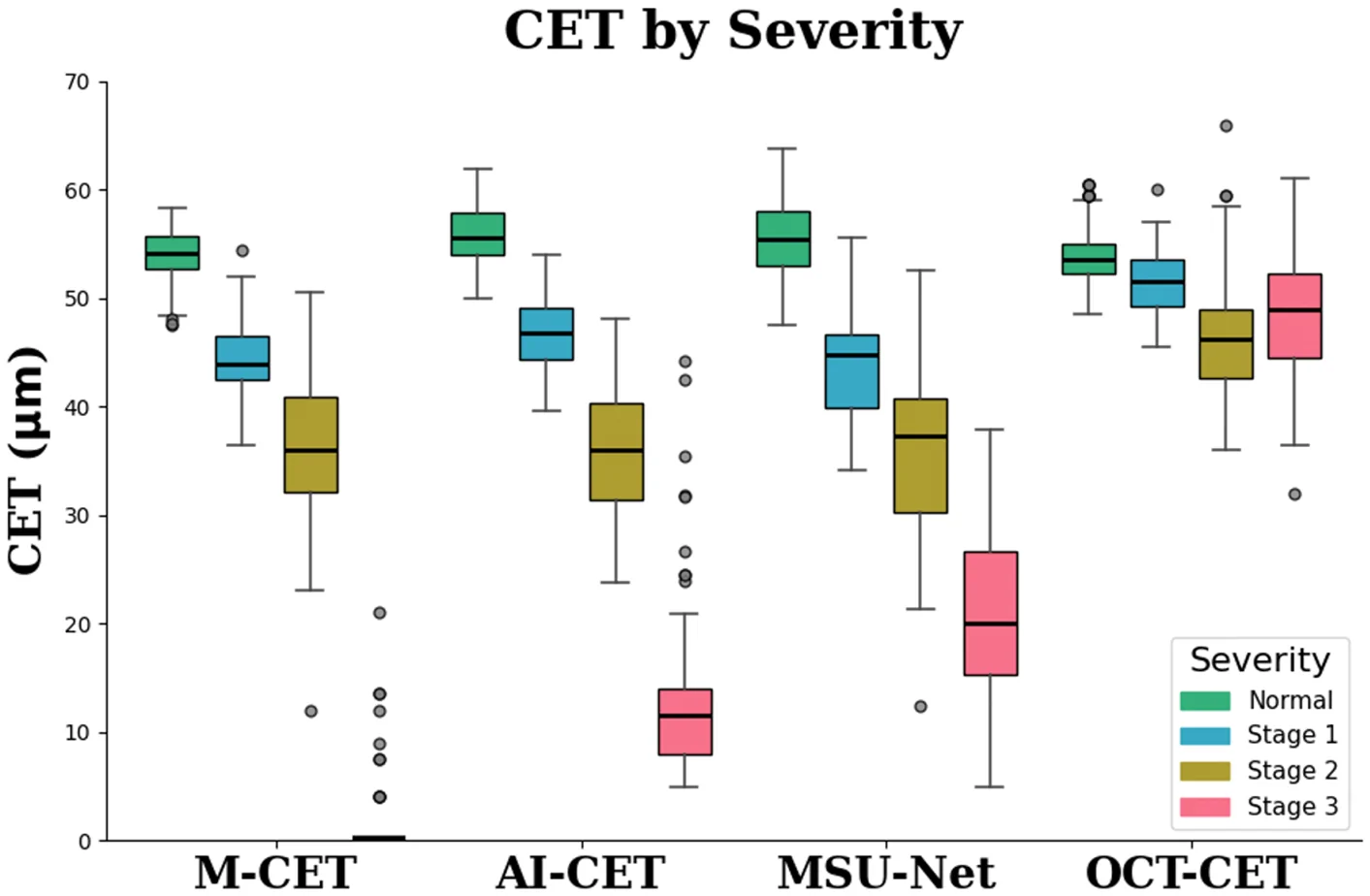
Primary dataset boxplots. Boxplots of predicted M-CET, AI-CET, MSU-Net, and OCT-CET values for normal, Stage 1, Stage 2, and Stage 3 scans.

Evaluating the baseline MSU-Net model by training on the original AS-OCT size images resulted in an overall significantly higher MAE (7.20 μm, p = 0.02) and an overall significantly lower correlation (r = 0.89, p = 0.01) than AI-CE. Differences in MAE and correlation were significant for the individual three LSCD severities (p < 0.001 for all three for both MAE and correlation), but not for normal (p = 0.21 for MAE, p = 0.19 for correlation). While MSU-Net did not perform as well as AI-CET, its overall MAE was still significantly lower (p < 0.001), and its overall correlation was still significantly higher (p < 0.001) than OCT-CET.

AI-CET stage classification distributions were not significantly different (p = 0.20) from those using manual measurement (Fig 4), with a comparable overall accuracy (70.7% vs. 70.6%) and improved significantly over OCT-CET (p < 0.001). Compared to M-CET, AI-CET had a slightly higher bias towards Stage 1 as it had a higher Stage 1 recall (0.660 vs. 0.531), but slightly lower recall for the other stages. Notably, classification using AI-CET was off by more than one stage fewer times than manual measurement (40 vs. 58). Comparatively, OCT-CET had a high bias towards the normal class, with the highest accuracy for normal, and decreasing accuracy as LSCD severity increased. OCT-CET did not correctly classify any Stage 3 images, instead placing most of these images into Stage 2.

**Fig 4:**
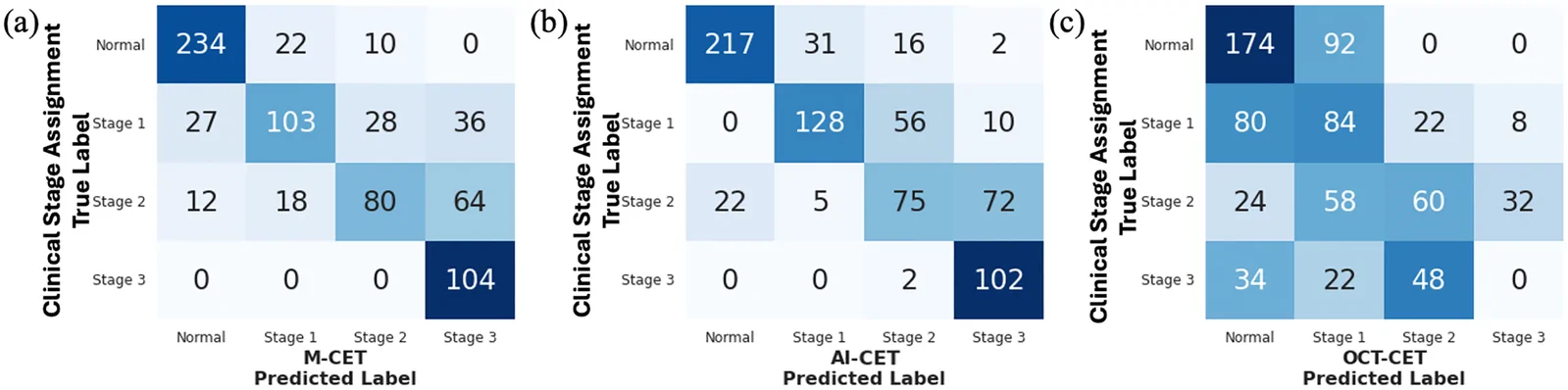
Primary dataset confusion matrices. Confusion matrices of the established clinical cut-off threshold true labels vs. the M-CET (a), AI-CET (b), and OCT-CET (c) predicted severity classes [2].

In the secondary retrospective dataset, AI-CET values correlated well with M-CET (r = 0.94, p < 0.001, Fig 5) while OCT-CET values did not (r = 0.37, p = 0.06) (Table 2). Overall MAE was significantly lower (p < 0.001) for AI-CET (MAE = 6.04 μm) compared to OCT-CET (MAE = 13.16 μm).

**Table 2:** MAE (mean and scan-level standard deviation) and Pearson correlation in the secondary retrospective set for the AI-CE and OCT-CET models.

Class	MAE (μm)			Pearson Correlation		
	All	Normal	Stage 3	All	Normal	Stage 3
**AI-CE**	**6.04 ± 4.10***	4.14 ± 3.57	**7.94 ± 4.57***	0.94*	0.94	0.79*
**OCT-CET**	13.16 ± 6.32	**3.98 ± 3.06**	22.34 ± 9.59	0.37	**0.96**	0.11

Note: *p < 0.01.

**Fig 5:**
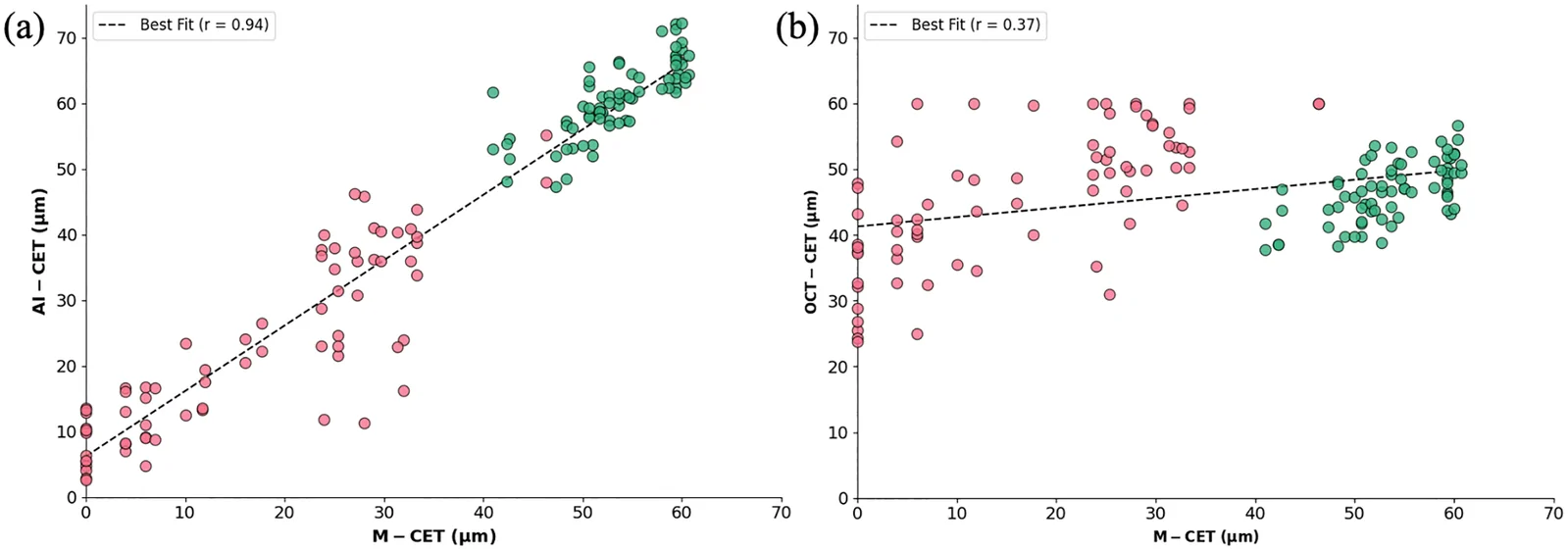
Secondary retrospective dataset correlations. Correlation of the secondary retrospective dataset for the AI-CE model compared to M-CET values (a) and the OCT-CET and M-CET values (b).

Qualitatively, the segmented epithelial layer produced by AI-CE was progressively thinner as LSCD severity increased (Fig 6), while OCT-CET did not demonstrate that trend. OCT-CET incorrectly segmented epithelial scar in 9.3% more cases than AI-CET. Comparatively, OCT-CET software always outputs a segmentation for each image across the entire cornea, even if there exists only subepithelial scar in the image. In all the Stage 3 images that contained subepithelial scar, regions of scarring were incorrectly included in the segmentation output by OCT-CET (Fig 7).

**Fig 6:**
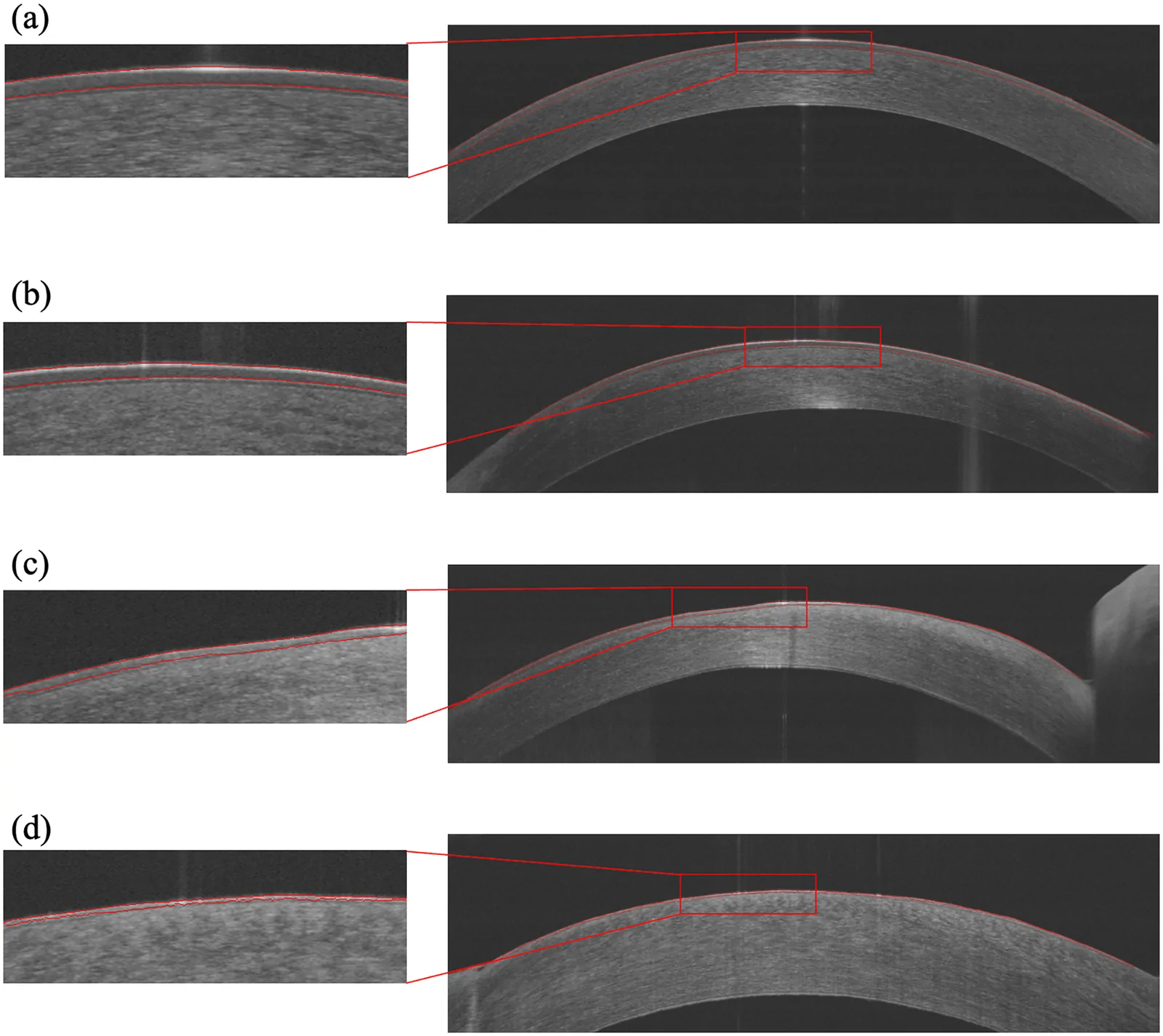
Corneal epithelial layer segmentations. Examples of AI-CE output segmentations of the epithelial layer for normal (a), Stage 1 (b), Stage 2 (c), and Stage 3 (d) LSCD.

**Fig 7:**
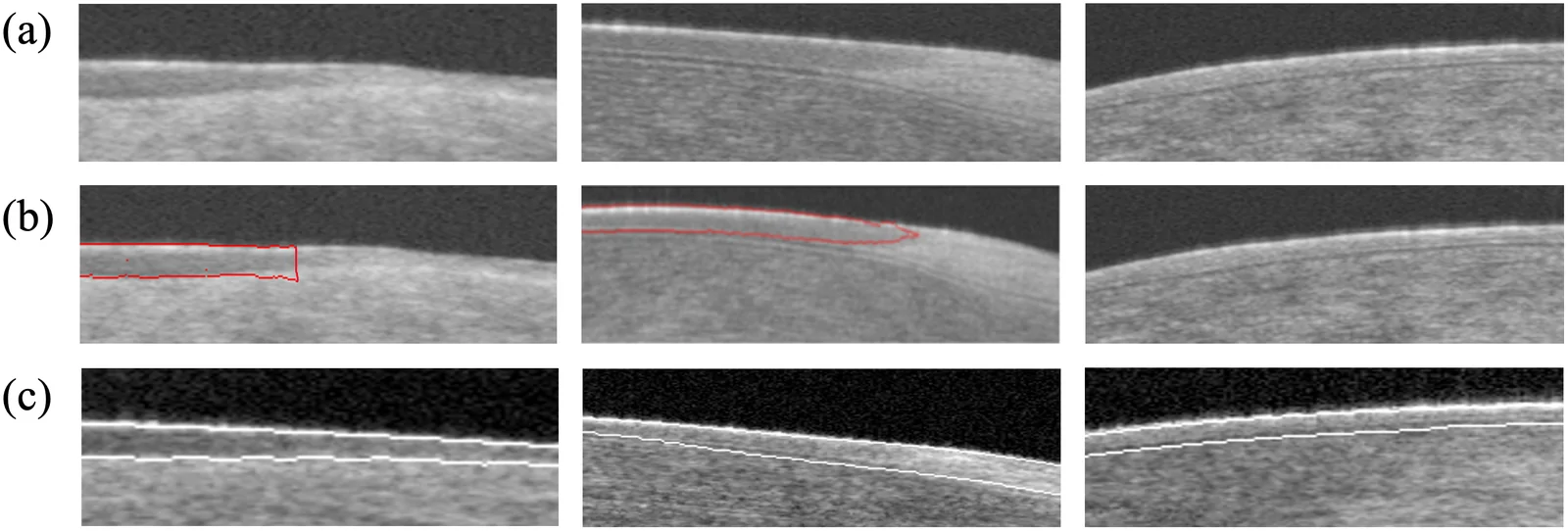
Stage 3 scarring segmentations. Examples of Stage 3 corneas that contain subepithelial scarring (a), output segmentations of the AI-CE model (b), and output segmentations of OCT-CET clinical software (c).

## Discussion

The AI-CE model showed significant improvement segmentation of the the corneal epithelium over exiting commercial software. The AI-CE model also allowed for improved LSCD severity prediction compared to commercial OCT-CET software. The similar LSCD stage classifications between AI-CET and cornea specialist labels suggest that the AI-CE model could potentially be used as an assistive device in the clinic by ophthalmologists. This tool would aid in the diagnostic process by emulating the clinical method of measuring CET, improving diagnostic efficiency and reducing workload.

The AI-CE model is able to more accurately classify Stage 1 eyes compared the manual measurements used in clinical practice. However, this improved classification is likely due to a difference in calibration as previously established thresholds are based on analysis of manual measurements [2]. Evidence of this calibration can be seen in normal patients, where the AI-CE model produces more false positive results. Thresholding on CET provided imperfect results whether using AI-CET or M-CET, which was a result of using only epithelial thickness for classification rather than applying the clinical practice of combining results with other scan modalities (slit-lamp biomicroscopy and in vivo laser scanning confocal microscopy) to determine diagnosis [2]. Additionally, most incorrect classifications by either model were off by a single stage, which may be due to a continuum in LSCD disease severity progression with overlap between stages.

Epithelial scarring can be easily confused with epithelium, which could explain why many Stage 3 images are predicted as having a high CET by OCT-CET. OCT-CET software is likely not trained with examples containing subepithelial scar and thus has no reference to distinguish subepithelial scar from healthy epithelium. OCT-CET also does not differentiate thickness across severities well, which could be because the software expects to find a thickness value within an established predictive range of values. In contrast, AI-CE is able to differentiate severities similarly to manual segmentations, which is likely due to the incorporation of the manual M-CET values when training the AI-CE model.

A benefit of the AI-CE model is its ability to obtain a segmentation across the full corneal surface, rather than the single CET value per cornea produced manually. This complete segmentation allows for capturing variations of epithelial thickness along the cornea. Analysis of this variation could lead to improved analysis of LSCD by allowing further examination of which areas of the cornea (beyond just the center) are thinning or contain regions of subepithelial scar.

The AI-CE model could potentially be used to help detect thinning of the epithelial layer in other corneal diseases, such as in keratoconus and myopia [30,31], or potentially aiding clinicians in localizing eye scarring when planning for operations such as refractive laser surgery. Segmentation of thinner epithelial layer caused by keratoconus could potentially be achieved by AI-CE, as it is functionally a similar task to segmenting the thinner epithelial layer caused by LSCD. Similarly, the model could be applied to segment and measure epithelial layer thickness differences in pre and post orthokeratology contact lens wear [32]. Similar ocular surface diseases that manifest with thinning or distortion of the epithelial layer would be suitable targets for segmentation by the AI-CE model.

### Future directions

Changes in epithelial reflectivity due to subepithelial scarring in Stage 3 images can cause difficulties in segmentation for the AI-CE model. If scar tissue is present, the AI-CE model could be given more knowledge in determining the boundaries of the epithelial layer by explicitly defining a classification task to determine epithelial layer versus scar tissue, guided by the average intensity (representing reflectivity) of the epithelial layer [2,3]. This approach would likely provide the model more certainty in localizing the bottom boundary of the epithelial layer, as the explicit encoding of reflectivity within the model would help guide its decisions in determining segmentation boundaries. The AI-CE model could also be applied to generate epithelial thickness maps over the corneal surface from predicted CET measurements. This approach could provide an improvement over maps currently provided by commercial software, especially for Stage 3 eyes.

## Conclusion

We proposed AI-CE, a DL model to measure CET in LSCD eyes within AS-OCT images. This model is an improvement over current clinically available software, providing measurements that are similar to manual measurements. Applying this model to the clinic could reduce clinical workload by reducing the need for manual annotations and corrections to commercial software. LSCD severity staging via this model can also be achieved by classifying patients based on the predicted measured CET, although future work will be required to show its performance as part of a multi-modal diagnosis. The similarity between the AI-CE model’s predicted CET values and manual measurements suggests potential for its use as an automated tool for segmenting and measuring the epithelial layer.

## Supporting information

S1 File CET values for the original dataset and the secondary retrospective data set. The individual CET values of the AI-CE, OCT-CET, and M-CET used to generate the correlation and box plots presented in the study.
(XLSX)

## References

[1] Deng SX, et al. Global consensus on definition, classification, diagnosis, and staging of limbal stem cell deficiency. Cornea. 2019;38(3):364–75. doi: 10.1097/ICO.0000000000001820 PMC6363877 30614902

[2] Le Q, Chauhan T, Cordova D, Tseng CH, Deng SX. Biomarkers of in vivo limbal stem cell function. Ocul Surf. 2022;23:123–30. doi: 10.1016/j.jtos.2021.12.005 34902592

[3] Liang Q, Le Q, Cordova DW, Tseng C-H, Deng SX. Corneal epithelial thickness measured using anterior segment optical coherence tomography as a diagnostic parameter for limbal stem cell deficiency. Am J Ophthalmol. 2020;216:132–9. doi: 10.1016/j.ajo.2020.04.006 32283095 PMC7434708

[4] Liu P, et al. MobileNetV2 Based Diagnosis and Grading of Limbal Stem Cell Deficiency. In: 2022 IEEE BIBE. Taichung, Taiwan; 2022. pp. 174–9.

[5] Gibson D, Tran T, Raveendran V, Bonnet C, Siu N, Vinet M, et al. Latent diffusion augmentation enhances deep learning analysis of neuro-morphology in limbal stem cell deficiency. Front Med (Lausanne). 2023;10:1270570. doi: 10.3389/fmed.2023.1270570 37908848 PMC10613638

[6] U.S. Food and Drug Administration website. Optovue Inc. iVue, 510(k) Summary. 2017. www.accessdata.fda.gov/cdrh_docs/pdf16/K163475.pdf

[7] Levy A, Georgeon C, Knoeri J, Tourabaly M, Leveziel L, Bouheraoua N, et al. Corneal Epithelial thickness mapping in the diagnosis of ocular surface disorders involving the corneal epithelium: a comparative study. Cornea. 2022;41(11):1353–61. doi: 10.1097/ICO.0000000000003012 35349542 PMC9555759

[8] Hao P, Shi K, Tian S, Wu F. Uncertainty‐aware iterative learning for noisy‐labeled medical image segmentation. IET Image Process. 2023;17(13):3830–40. doi: 10.1049/ipr2.12900

[9] Peng J, Wang Y. Medical image segmentation with limited supervision: a review of deep network models. IEEE Access. 2021;9:36827–51. doi: 10.1109/access.2021.3062380

[10] Athanasiou G, Arcos JL, Cerquides J. Enhancing medical image segmentation: ground truth optimization through evaluating uncertainty in expert annotations. Mathematics. 2023;11(17):3771. doi: 10.3390/math11173771

[11] Theodoridis J, Hofmann J, Maucher J, Schilling A. Trapped in texture bias? A large scale comparison of deep instance segmentation. In: Lecture Notes in Computer Science; 2022. pp. 609–27.

[12] Qin W, Wu J, Han F, Yuan Y, Zhao W, Ibragimov B, et al. Superpixel-based and boundary-sensitive convolutional neural network for automated liver segmentation. Phys Med Biol. 2018;63(9):095017. doi: 10.1088/1361-6560/aabd19 29633960 PMC5983385

[13] Xu R, Xu C, Li Z, Zheng T, Yu W, Yang C. Boundary guidance network for medical image segmentation. Sci Rep. 2024;14(1):17345. doi: 10.1038/s41598-024-67554-0 39069513 PMC11284230

[14] Zhao L, Wang T, Chen Y, Zhang X, Tang H, Lin F, et al. A novel framework for segmentation of small targets in medical images. Sci Rep. 2025;15(1):9924. doi: 10.1038/s41598-025-94437-9 40121297 PMC11929788

[15] Sang S, Zhou Y, Islam MT, Xing L. Small-object sensitive segmentation using across feature map attention. IEEE Trans Pattern Anal Mach Intell. 2023;45(5):6289–306. doi: 10.1109/TPAMI.2022.3211171 36178991 PMC10823909

[16] Konrad PM, Popa AA, et al. Challenges in Deep Learning-Based Small Organ Segmentation: A Benchmarking Perspective for Medical Research with Limited Datasets. 2026. doi: 10.48550/arXiv.2509.05892

[17] Ronneberger O, Fischer P, Brox T. U-Net: Convolutional Networks for Biomedical Image Segmentation. Lecture Notes in Computer Science. Springer International Publishing; 2015. pp. 234–41. doi: 10.1007/978-3-319-24574-4_28

[18] Dos Santos VA, Schmetterer L, Stegmann H, Pfister M, Messner A, Schmidinger G, et al. CorneaNet: Fast segmentation of cornea OCT scans of healthy and keratoconic eyes using deep learning. Biomed Opt Express. 2019;10(2):622–41. doi: 10.1364/BOE.10.000622 30800504 PMC6377876

[19] Pham TH, Devalla SK, Ang A, Soh Z-D, Thiery AH, Boote C, et al. Deep learning algorithms to isolate and quantify the structures of the anterior segment in optical coherence tomography images. Br J Ophthalmol. 2021;105(9):1231–7. doi: 10.1136/bjophthalmol-2019-315723 32980820

[20] Li K, Yang G, Chang S, Yao J, He C, Lu F, et al. Comprehensive assessment of the anterior segment in refraction corrected OCT based on multitask learning. Biomed Opt Express. 2023;14(8):3968–87. doi: 10.1364/BOE.493065 37799701 PMC10549746

[21] Goyanes E, Moura J de, Fernández-Vigo JI, Fernández-Vigo JA, Novo J, Ortega M. Automatic simultaneous ciliary muscle segmentation and biomarker extraction in AS-OCT images using deep learning-based approaches. Biomed Signal Process Control. 2024;90:105851. doi: 10.1016/j.bspc.2023.105851

[22] Fang J, Xing A, Chen Y, Zhou F. SeqCorr-EUNet: A sequence correction dual-flow network for segmentation and quantification of anterior segment OCT image. Comput Biol Med. 2024;171:108143. doi: 10.1016/j.compbiomed.2024.108143 38364662

[23] Win KY, Fai JWH, Ying WQ, Qi CCS, Chua J, Wong D, et al. Corneal layer segmentation in healthy and pathological eyes: A joint super-resolution generative adversarial network and adaptive graph theory approach. Transl Vis Sci Technol. 2025;14(3):19. doi: 10.1167/tvst.14.3.19 40105812 PMC11932425

[24] Elsawy A, Gregori G, Eleiwa T, Abdel-Mottaleb M, Shousha MA. Pathological-corneas layer segmentation and thickness measurement in OCT images. Transl Vis Sci Technol. 2020;9(11):24. doi: 10.1167/tvst.9.11.24 33173606 PMC7594599

[25] Goto H, Ono T, Taketani Y, Abe Y, Kimakura M, Toyono T, et al. Corneal irregularity of the anterior and posterior surface in patients with limbal stem cell deficiency evaluated with anterior-segment optical coherence tomography. Jpn J Ophthalmol. 2025;69(5):708–16. doi: 10.1007/s10384-025-01242-y 40690095 PMC12391154

[26] Greenfield JA, Scherer R, Alba D, De Arrigunaga S, Alvarez O, Palioura S, et al. Detection of ocular surface squamous neoplasia using artificial intelligence with anterior segment optical coherence tomography. Am J Ophthalmol. 2025;273:182–91. doi: 10.1016/j.ajo.2025.02.019 39983942 PMC11985264

[27] Kittipibul T, et al. Comparison of automated and manual measurement of corneal epithelial thickness in eyes with limbal stem cell deficiency. Cornea. 2025;44(4):422–6. doi: 10.1097/ICO.0000000000003682 PMC11865354 39196902

[28] Ye Y, et al. DiffusionEdge: Diffusion Probabilistic Model for Crisp Edge Detection. In: 2024 AAAI Conference on Artificial Intelligence. Vancouver, Canada; 2024. doi: 10.48550/arXiv.2401.02032

[29] Su R, Zhang D, Liu J, Cheng C. MSU-Net: Multi-Scale U-Net for 2D Medical Image Segmentation. Front Genet. 2021;12:639930. doi: 10.3389/fgene.2021.639930 33679900 PMC7928319

[30] Mlyniuk P, Kaszuba-Modrzejewska M, Rzeszewska-Zamiara J, Piotrowiak-Slupska I, Kaluzny BJ. Corneal epithelial thickness maps in eyes with mild and moderate keratoconus. J Clin Med. 2023;14(4):1256. doi: 10.3390/jcm14041256 PMC11856637 40004787

[31] Nemoto K, Kamiya K, Hata S, Kobayashi M. Post-orthokeratology changes in the corneal epithelium induced by an orthokeratology lens: An anterior-segment optical coherence tomography assessment. Eye Contact Lens. 2016;52(5):198–202. doi: 10.1097/ICL.0000000000001267 PMC13102347 41888630

[32] Zhang J, Li J, Li X, Li F, Wang T. Redistribution of the corneal epithelium after overnight wear of orthokeratology contact lenses for myopia reduction. Contact Lens Anterior Eye. 2020;43(3):232–7. doi: 10.1016/j.clae.2020.02.015 32127287

